# Pancreatic Surgery for Cystic Lesions in Elderly Patients: Is Age a Relevant Risk Factor for Postoperative Complications?

**DOI:** 10.7759/cureus.115660

**Published:** 2026-09-02

**Authors:** Fabian Nimczewski, Manuela Kruegermeyer-Kalthoff, Sjaak Pouwels, Krzysztof Nowakowski, Kira Baginski, Annika Hoyer, Jens Hoeppner, Zsolt Madarasz

**Affiliations:** 1 Surgery, Medical School and University Medical Center OWL, Campus Lippe, Bielefeld University, Detmold, DEU; 2 General and Abdominal Surgery, Bielefeld University, Bielefeld, DEU; 3 Biostatistics and Medical Biometry, Bielefeld University, Bielefeld, DEU

**Keywords:** elderly patients, pancreatic cyst, pancreatic fistula, pancreatic surgery, postoperative complications

## Abstract

Background

The increasing use of high-resolution imaging techniques in an aging society leads to an increased number of cystic pancreatic lesion diagnoses. In this study, we compared two patient groups who underwent surgery for a benign pancreatic cystic lesion, investigating whether a difference in postoperative outcomes can be observed based on patient age.

Methods

The single-center retrospective analysis included 54 patients who underwent surgery for a benign pancreatic cystic lesion between 2015 and 2024. Two groups were formed. Group I included 26 patients who were 70 years or younger at the time of surgery, and Group II consisted of 28 patients who were older than 70 years. The primary endpoint was 90-day mortality. Secondary endpoints were the risk of postoperative pancreatic fistulas, the revision rate, surgical site infection, delayed gastric emptying, ventilation time longer than 48 hours, need for postoperative transfusion, and the occurrence of postoperative pneumonia.

Results

A higher 90-day mortality was observed in the older patient group (14.3% versus 7.7%, p = 0.562). A clinically relevant difference was found in the risk of postoperative pancreatic fistulas (30.8% versus 3.6%, p = 0.028). The other secondary endpoints pneumonia risk (7.7% versus 10.7%, p = 0.254), pancreatic anastomosis insufficiency risk (11.1% versus 12.5%, p = 0.244), surgical site infection risk (7.7% versus 3.6%, p = 0.738), delayed gastric emptying (15.4% versus 14.3%, p = 0.998), postoperative ventilation time over 48 hours (11.5% versus 14.3%, p = 0.520), revision rate (23.1% versus 17.9%, p = 0.666), postoperative bleeding requiring transfusion (15.4% versus 7.1%, p = 0.814) and postoperative length of stay (17.3 days versus 17.4 days, p = 0.441) did not differ relevantly between the groups.

Conclusion

Based on our study, there were statistically significant differences in the risk of postoperative pancreatic fistulas and higher mortality in the older patient group. Based on the retrospective nature, definitive claims can not be made.

## Introduction

Due to the increasing use of high-resolution imaging techniques and demographic changes with an aging society, pancreatic cysts are increasingly being diagnosed in older patients. Zerboni et al. [1] conducted a systematic review and meta-analysis, which included a total of 48860 patients, and they found a pooled prevalence of 8% of incidentally diagnosed pancreatic cystic lesions (ranging from 0.2% to 45.9%). Dividing the sample into two age groups, they found a prevalence of 11% in patients aged 55 years and older, compared with 5.7% in the younger group [1]. Although a large proportion of pancreatic cysts remain asymptomatic and unsuspicious, certain cysts can develop into malignancy over time. The challenge lies in identifying those cysts with relevant clinical, diagnostic, and histologic risks and scheduling surgical resection at the appropriate time point for these patients. Del Chiaro et al. [2] conducted a retrospective cohort study, and they tried to correlate the preoperative diagnostics with the postoperative histological findings, e.g., diagnosis. They found that in 141 patients, the overall accuracy of preoperative diagnostics was 60.9% [2]. They also found that the overall accuracy did not significantly differ between asymptomatic and symptomatic patients [2].

Pancreatic cysts represent a heterogeneous entity. Preoperative diagnosis is particularly inaccurate for cysts under 2 cm. However, accurate prediction is crucial, especially given the widely varying risk of malignancy. The range extends from a 0% risk of malignancy for dysontogenic cysts or pseudocysts up to a 66% risk of malignancy for some main duct intraductal papillary mucinous neoplasm (IPMN) [3]. Since pancreatic cysts represent a precancerous condition and can degenerate over time, increasing patient age is a risk factor that must be taken into account [4]. Pancreatic cysts with a potential risk of malignancy include mucinous cystic neoplasm (MCN) and solid pseudopapillary neoplasm (SPN), each with a 10-15% risk of malignancy, as well as IPMN. In IPMN, the risk of malignancy depends on whether there is a connection to the main pancreatic duct (MD-IPMN) or a branch duct (BD-IPMN) [4]. In MD-IPMN, the risk of malignancy ranges from 33 to 66% [3]. In contrast, the risk of malignancy in BD-IPMN is reported to be 2 to 10%. The typical age for onset of MCN is 40 to 50 years, and it affects almost exclusively women [3]. The peak age for SPN is 20 to 40 years. In older patients, IPMN is particularly relevant, with a typical age of onset of 60 to 80 years [5].

Our study aims to investigate whether age could be a determining factor in patients who underwent resection for pancreatic cysts. We hypothesize that older age groups are more prone to complications compared to younger patients because of the presence of more comorbidities.

## Materials and methods

This study was reported in accordance with the Strengthening the Reporting of Observational Studies in Epidemiology (STROBE) guideline [6] and adheres to the principles laid down in the 1964 Declaration of Helsinki.

Patient population and data collection

This study included patients who underwent surgery for a benign cystic pancreatic lesion between 2015 and 2024 at the Department of Surgery, Medical School and University Medical Center OWL, Campus Lippe, Bielefeld University, Detmold, Germany.

The total group of patients with pancreatic surgery for benign pancreatic lesions was categorized into two subgroups, based on a median age of 70 years in the total patient population. Patients with evidence of malignancy and operations due to chronic pancreatitis were excluded. The resection procedures were pancreatic head resections, left pancreatic resections, and parenchyma-sparing enucleations. All operations were performed by certified center surgeons or assisted by them. Since 2024, minimally invasive techniques have also been increasingly used. The patient data were pseudonymized and transferred to an internal pancreas registry.

Statistical analysis

Due to the retrospective nature of this study, a sample size calculation was not conducted. As stated before, the division into the two subgroups was based on the calculated median age of 70 years of the total study population (n =54). Initially, patient characteristics were assessed descriptively, employing mean (standard deviation) for continuous variables and percentage for categorical variables.

For binary outcomes, multivariable logistic regression was performed, while for continuous outcomes, multivariable linear regression was used. In both models, age group, sex, body mass index (BMI), and smoking status were included as covariates. Effect estimates were reported as odds ratios (ORs) with 95% confidence intervals (CIs) for logistic regression, and as regression coefficients with 95% CIs for linear regression. A p-value ≤ 0.05 was considered statistically significant. R software (version 4.5.2; R Foundation for Statistical Computing, Vienna, Austria) was used to prepare the database and for statistical analysis.

## Results

Patient characteristics

A total of 318 pancreatic resections were performed at our Center for Pancreatic Surgery during the analysed period. A total of 54 patients had a benign cystic pancreatic lesion. The procedure for patient selection is shown in Figure 1. Twenty-six patients were 70 years of age or younger at the time of surgery, and 28 patients were older than 70 at the time of surgery.

**Figure 1:**
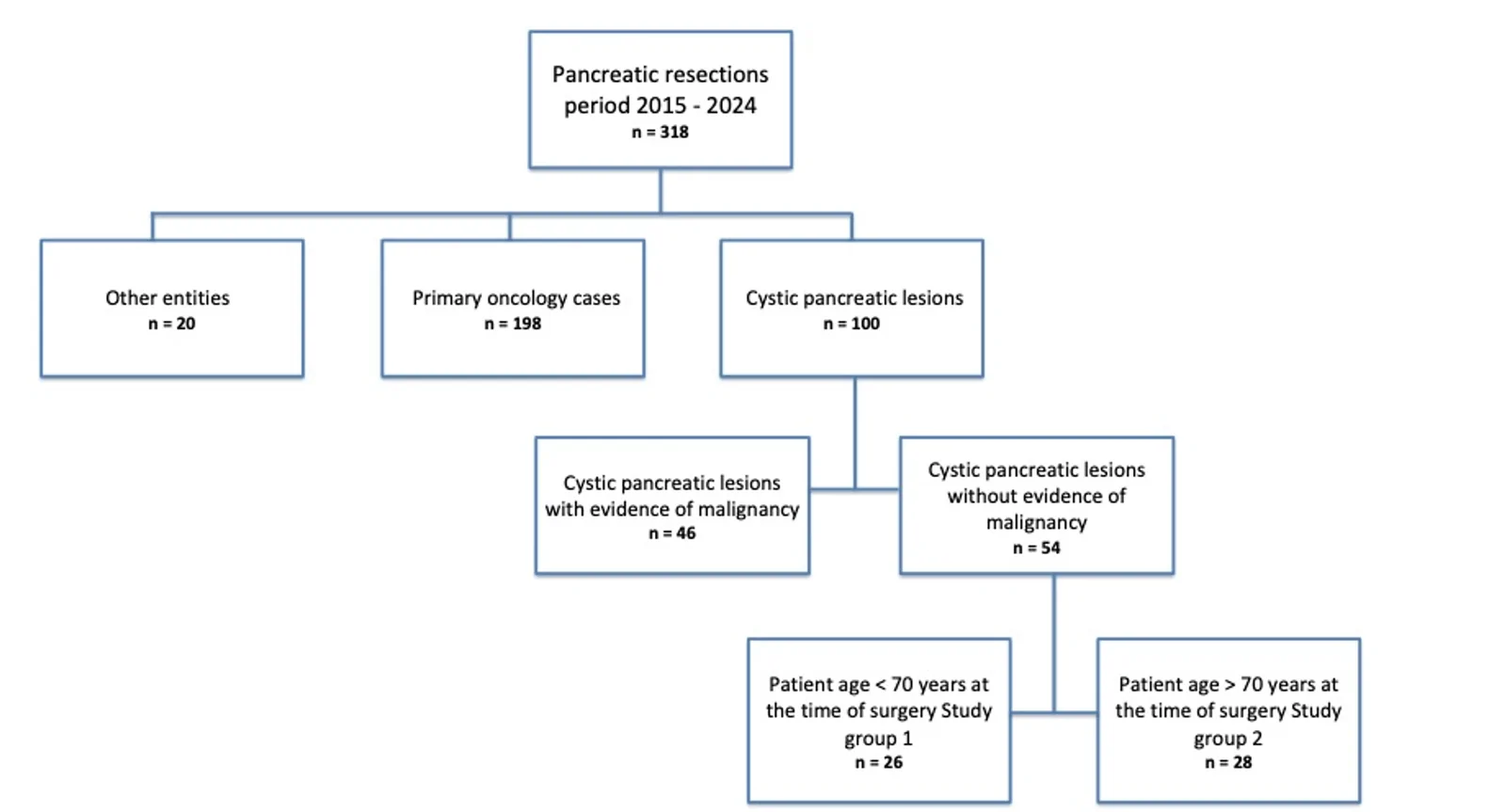
Flowchart of patient selection

Group I had younger patients, with an average age of 62.9 ± 8.13 years, than Group II, with an average age of 76.5 ± 3.43 years. There were no relevant differences between the groups regarding other baseline parameters such as sex, BMI, American Society of Anesthesiologists (ASA) score, and Eastern Cooperative Oncology Group (ECOG) grade (Table 1).

**Table 1:** General patient characteristics SD: standard deviation; BMI: body mass index; ASA: American Society of Anesthesiologists; ECOG: Eastern Cooperative Oncology Group

Characteristics	Group I (N = 26)	Group II (N = 28)	Overall (N = 54)
Sex			
Male	11 (42.3%)	12 (42.9%)	23 (42.6%)
Female	15 (57.7%)	16 (57.1%)	31 (57.4%)
Age (in years)			
Mean (SD)	62.9 (8.13)	76.5 (3.43)	69.9 (9.17)
BMI			
Mean (SD)	26.6 (5.00)	25.3 (4.15)	25.9 (4.58)
ASA score			
1	2 (7.7%)	3 (10.7%)	5 (9.3%)
2	20 (76.9%)	22 (78.6%)	42 (77.8%)
3	4 (15.4%)	3 (10.7%)	7 (13.0%)
ECOG classification			
0	24 (92.3%)	25 (89.3%)	49 (90.7%)
1	2 (7.7%)	3 (10.7%)	5 (9.3%)

Surgery details

In Group I, nine pylorus-preserving pancreatic head resections, six distal pancreatic resections, and five enucleations were performed. In this group, six revisional operations were performed. In Group II, 11 pancreatic head resections, 10 distal pancreatic resections, and two pancreatic segment resections were performed. In this group, a total of five revisional procedures were performed. A total of four operations were performed using minimally invasive techniques: two operations in Group I and two operations in Group II. These included two parenchyma-sparing enucleations in Group I and two distal pancreatectomies in Group II. The other 50 resections were performed using open technique.

Postoperative outcomes and complications

The postoperative outcomes, including complications and postoperative length of stay, are presented in Tables 2-3. Although the odds of 90-day mortality were greater in Group II than in Group I (OR 1.75; 95% CI 0.28-14.8), the difference was not statistically significant (p = 0.562). Patients in Group II experienced a 92% reduction in the odds of pancreatic fistulas compared with Group I, which was statistically significant (OR 0.08; 95% CI 0.00-0.55; p = 0.028).

**Table 2:** Postoperative outcomes and complications OR: odds ratio (Group II vs. Group I); CI: confidence interval; POPF: postoperative pancreatic fistula; SSI: surgical site infection; DGE: delayed gastric emptying

	Group I (N = 26)	Group II (N = 28)	OR	95% CI	P-value
90-day mortality	2 (7.7%)	4 (14.3%)	1.75	(0.28, 14.8)	0.562
POPF	8 (30.8%)	1 (3.6%)	0.08	(0.00, 0.55)	0.028
Revision	6 (23.1%)	5 (17.9%)	1.42	(0.30, 8.01)	0.666
SSI	2 (7.7%)	1 (3.6%)	0.63	(0.02, 10.4)	0.738
DGE	4 (15.4%)	4 (14.3%)	1.00	(0.19, 5.39)	0.998
Ventilation >48 hours	3 (11.5%)	4 (14.3%)	1.85	(0.30, 15.5)	0.520
Transfusion	4 (15.4%)	2 (7.1%)	0.78	(0.08, 7.45)	0.814
Pneumonia	2 (7.7%)	3 (10.7%)	4.44	(0.44, 112)	0.254

**Table 3:** Postoperative length of stay (in days) Values are presented as mean (SD). CI: confidence Interval; SD: standard deviation; ICU: intensive care unit

	Group I (N = 26)	Group II (N = 28)	Estimate	95% CI	P-value
ICU	3.73 (5.38)	5.25 (6.92)	1.9	(-1.8, 5.6)	0.315
Inpatient hospital stay	17.3 (8.49)	17.4 (8.48)	1.9	(-3.0, 6.8)	0.441

In Group I, there were six pancreatic fistulas of type A, one type B, and one type C (as defined by the 2016 Update of the International Study Group (ISGPS) [7]. In Group II, there was one type B fistula. The secondary endpoints of revision rate, surgical site infection, delayed gastric emptying, duration of ventilation over 48 hours postoperatively, transfusion rate, and number of postoperative pneumonias showed no significant difference between the groups. On average, both the length of intensive care stay and the length of hospital stay in Group II were 1.9 days longer than in Group I, after adjusting for other covariates (age, sex, BMI, and smoking status). However, these differences were not statistically significant (95% CI: -1.8 to 5.6; p = 0.315 for ICU stay and 95% CI: -3.0 to 6.8; p = 0.441 for hospital stay).

## Discussion

In this single-center retrospective analysis, the postoperative outcomes of 54 patients who underwent pancreatectomy for a benign pancreatic cyst within a 10-year interval were evaluated, stratified by patient age. We found statistically significantly higher odds of postoperative pancreatic fistula (POPF) in patients who were younger than 70 years of age. For younger patients, the question of whether to undergo pancreatic surgery is crucial in terms of invasiveness and the potential occurrence of complications. For older patients, the risk of malignant transformation is weighed against the risk of a complicated pancreatic operation in relation to life expectancy.

It may be considered that there might be different postoperative complication profiles between patients who undergo pancreatic surgery for a benign lesion compared to a malignant lesion. Also, the extent of the pancreatic surgery (for example, pancreatectomy versus enucleation) must be considered. For patients who underwent surgery for pancreatic ductal adenocarcinoma, there is already data available. In an analysis of the German StuDoQ data, patient age is described as a significant influencing factor on perioperative outcomes after pancreatic resections for pancreatic cancer: no statistically significant difference in pancreatic surgery-specific complications among age groups was found. Patients with late-onset pancreatic cancer had an overall higher risk of complications, with substantially more complications graded Clavien-Dindo >3a among patients who underwent pancreaticoduodenectomy. Among patients who underwent distal pancreatectomy, there was a trend toward higher mortality; however, the difference was not statistically significant between the different onset types of pancreatic cancer [5]. So, based on these study results, the postoperative course of patients after pancreatic surgery is influenced by a variety of factors, including the age of onset of the pancreatic cancer. These findings were supported by the meta-analysis of Tan et al. They showed that the patient’s age could not be used as the single predictive factor for assessing the risk of postoperative complications after pancreatic resection in patients with malignant tumours [8]. Similar results were found in the retrospective study done by Wang et al. They found that age had no significant influence on the postoperative course and occurrence of complications, or on long-term survival after pancreatoduodenectomy. In the same study, it was shown that the presence of cardiovascular comorbidities or poor preoperative nutritional status was significantly associated with a higher risk of complications and even mortality [9].

Interestingly, we have found in our current study that the odds of POPF were statistically significantly higher in patients younger than 70 years of age. Similar results were reported by Sahakyan et al. They studied 402 patients who underwent minimally invasive left pancreatectomy, of whom 118 (29.4%) were older than 70 years. The occurrence of POPF was significantly lower in the elderly patient group (18.6% versus 36.6%, p < 0.05) [10].

In the last few years, multiple attempts have been made to develop prediction models for POPF. Multiple factors have been identified that contribute to the occurrence of POPF, such as anatomical and biological characteristics. Among these, pancreatic tissue texture and the diameter of the main pancreatic duct are considered the most important factors [11]. The number of acinar cells at the resection margins of the pancreatic tissue has been identified as an accurate and objective predictor for POPF [12]. Similarly, pancreatic thickness and a greater number of stump cross-sections are reliable predictors for POPF [11,12]. Furthermore, patient-related factors like sarcopenic obesity, tumour characteristics, preoperative diabetes, surgical characteristics (operative time, blood loss, abdominal wall thickness) and neoadjuvant therapy have been associated with the occurrence of POPF [11,13].

When developing a prediction model using different dependent and independent risk factors, internal and external validation of such models can be challenging. This has been seen over the years since multiple prediction models have been developed. Unfortunately, these prediction models have had severe issues like insufficient reporting, inconsistent or optimistic methodologies, high risk of bias, and poor clinical usability and reproducibility [14]. In the coming years, clinicians should focus on developing an adequate model for prediction of POPF. It should be stated that artificial intelligence could be used to optimize the currently available prediction models or develop a completely new one. Similar attempts have been conducted in other specialties [15].

It can be stated that the postoperative course of patients who underwent pancreatic surgery is dependent on multiple patient, surgical, and diagnostic characteristics. In both malignant and benign lesions, careful consideration should be given to patients' age and clinical characteristics. Most of the literature nowadays is focused on malignant lesions, and therefore adequate data on pancreatic surgery for benign lesions is lacking. Especially in patients with cystic pancreatic lesions, patient age is a decisive argument for or against surgery, as the indication for surgery is often relative. Therefore, it is of the utmost importance that the preoperative risk assessment is optimal at the patient level (optimization of nutritional status and/or comorbidities), but also the risk of malignant transformation (based on the available diagnostics) should be considered in the "shared-decision-making" process. In today’s day and age, the availability of minimally invasive and increasingly robot-assisted procedures brings a clear advantage, which lies in the reduced tissue damage and faster recovery of patients [16]. These aspects will influence future study outcomes. Van der Heijde et al. [17] showed that minimally invasive pancreatic surgical procedures offer an advantage in elderly patients regarding intraoperative blood loss and length of hospital stay.

Due to the retrospective study design, the long study period, the high proportion of open resections and the small number of cases, and the potential influence of multiple factors and comorbidities, the present study results hamper generalizability. It needs to be taken into account that the small sample of patients could indicate not enough statistical power to detect potential differences. This is seen in the higher mortality in the group of patients older than 70 years (despite not being statistically significant), but also in the relatively wide CIs of the given ORs.

## Conclusions

Based on the data from this study, we have found a significantly higher rate of POPFs in patients younger than 70 years. Due to the retrospective study design, long follow-up period, and small sample size, this study serves as hypothesis-generating, and prospective, multicenter registry data are needed to support this hypothesis.
